# Prognostic and diagnostic value of PVR gene and protein levels, serum amylase, and urinary IGFBP-7 and TIMP-2 biomarkers in multiple myeloma

**DOI:** 10.1186/s12885-025-14241-6

**Published:** 2025-05-14

**Authors:** Eman M. Habib, Asmaa M. Hasan, Sara A. A. Mohammed, Amira A. A. Othman, Rasha Elgamal, Dalia E. Sherief

**Affiliations:** 1 Clinical Pathology Department Faculty of Medicine, Kafr Elsheikh University, Kafr Elsheikh, Egypt; 2 Oncology Department Faculty of Medicine, Kafr Elsheikh University, Kafr Elsheikh, Egypt; 3 Internal Medicine Department, Faculty of Medicine, Suez University, Suez, Egypt; 4 Clinical Pathology Department Faculty of Medicine, Suez University, Suez, Egypt

**Keywords:** Multiple myeloma, PVR gene expression, Serum amylase, Prognostic biomarkers, IGFBP-7, TIMP-2, Survival analysis, Risk stratification

## Abstract

**Background:**

Multiple Myeloma (MM) is a plasma cell malignancy associated with systemic and renal complications. This study evaluates the prognostic and diagnostic significance of poliovirus receptor (PVR) gene expression and protein levels, serum amylase, and urinary biomarkers (IGFBP-7, TIMP-2) in MM patients.

**Methods:**

In a prospective case-control study, 50 MM patients and 50 healthy controls were assessed. PVR gene expression (qPCR), serum PVR and amylase (ELISA/chemistry analyzer), and urinary IGFBP-7 and TIMP-2 (ELISA) were analyzed. Statistical analyses included correlation tests, Kaplan-Meier survival analysis, Cox regression, stratified quartile analysis, and receiver operating characteristic (ROC) curve evaluation. Multiple testing corrections (Bonferroni and FDR) were applied.

**Results:**

MM patients showed significantly elevated PVR expression and protein levels, serum amylase, and urinary biomarkers compared to controls (*p*<0.001). High PVR expression was associated with advanced disease stage, TP53 mutations, and reduced overall survival (OS: 44.84 vs. 48.0 months; *p*=0.044). High serum amylase and urinary IGFBP-7 were linked to significantly poorer OS and progression-free survival (PFS). Multivariate Cox regression confirmed PVR expression (HR=12.2), serum amylase (HR=11.5), and IGFBP-7 (HR=11.9) as independent predictors of poor OS, findings that remained robust in bootstrapped and penalized regression models. Stratified analysis revealed that patients in the highest biomarker quartiles had significantly worse outcomes and higher TP53 mutation rates. ROC analysis showed excellent diagnostic performance for the combined panel (PVR + amylase + IGFBP-7; AUC=0.97, sensitivity =90%, specificity = 88%), outperforming individual markers. Significant associations remained after multiple testing correction.

**Conclusion:**

PVR gene expression, serum amylase, and urinary IGFBP-7 are independent and robust prognostic biomarkers in MM. Their combined use enhances diagnostic accuracy and risk stratification, supporting their integration into clinical decision-making. Validation in larger, multi-center studies is recommended**.** Limitations include the single-center design, modest sample size, absence of disease comparator groups, and the cross-sectional nature of biomarker evaluation. These findings warrant validation in larger, multi-institutional, and longitudinal studies.

## Introduction

Multiple myeloma (MM), also known as plasma cell myeloma and simply myeloma, is a hematologic malignancy of plasma cells that accounts for approximately 10% of hematologic cancers and 1% of all cancers globally [1]. After non-Hodgkin lymphoma, it represents the second most common hematologic malignancy [2]. MM is characterized by the pernicious proliferation of monoclonal plasma cells in the bone marrow causing destructive bone lesions, renal failure, anemia, and hypercalcemia (CRAB criteria) [3]. The diagnosis of MM following the International Myeloma Working Group (IMWG) criteria, requiring ≥10% clonal bone marrow plasma cells or a biopsy-proven plasmacytoma plus evidence of one or more multiple myeloma defining events (MDE): CRAB features (hypercalcemia, renal failure, anemia, bone lesions), specific biomarkers such as ≥60% monoclonal plasma cells, serum-free light chain ratio ≥100, and multiple localized lesions on magnetic resonance imaging (MRI) [4]. Advanced diagnostic biomarkers have significantly improved the detection and classification of MM. However, the identification of valid, affordable prognostic biomarkers remains one of the biggest challenges, indicating a need for better methods of risk stratification and management [2].

The"Poliovirus receptor"(PVR), also known as CD155, is a crucial glycoprotein in the nectin-like protein family, playing a very important role in immune responses and diseases such as multiple myeloma [5]. PVR was initially identified as the receptor for the human poliovirus. Recently, several functions have also been uncovered. It acts as a cell adhesion protein that helps transendothelial leukocytes move across blood vessel walls by interacting with CD226 and TIGIT, proteins found on leukocytes [6]. As PVR is differentially regulated in a broad spectrum of cancers, overexpression of PVR has been reported in several malignancies, suggesting its possible use as a prognostic biomarker. PVR is upregulated in tumor development, enhancing tumor proliferation, migration, and invasiveness. It suppresses the antitumor function of T lymphocytes and NK cells via tumor-infiltrating myeloid cells and, as a result, suppresses antitumor immunity [7]. Furthermore, it has been observed that PVR upregulation is related to an increased metastatic potential of tumor cells. Understanding the role of PVR will provide targeted therapies and personalized treatments for malignancies, including multiple myeloma [8, 9].

Serum amylase is primarily produced by the pancreas and salivary glands. Its main function is to break down starches into sugars, aiding digestion. In a healthy individual, there is a net balance between amylase production from the pancreas (P-isoamylase: 40%) and salivary gland (S-isoamylase 60%) and clearance from kidneys or reticuloendothelial system [10]. In MM patients, elevated serum amylase levels are typically due to three mechanisms: macroamylasemia, where amylase forms complex macromolecules whose large size prevents its urinary excretion; ectopic production by tumor cells, which can synthesize amylase; and renal failure, which reduces the kidneys'ability to filter and remove amylase from the bloodstream [11]. Serum amylase is one of the easily available tests at cheaper rates as compared to other conventional markers. Thus, it has been considered an adequate prognostic tool even in economically backward conditions. Because of cost-effectiveness and wide availability, serum amylase can also be a good marker in monitoring disease prognosis in a resource-poor setting [12].

Multiple Myeloma is accompanied by renal dysfunction as one of the most common complications that can be caused either by excess immunoglobulins that are nephrotoxic or some other causes like hypercalcemia, infection, etc., and is associated with poor prognosis, particularly when progressive. Detecting kidney injury as early as possible is vital, but challenging, for disease control and restoration of renal function [13]. Urinary biomarkers like Urinary Insulin-Like Growth Factor-Binding Protein 7 (IGFBp7) and urinary Tissue Inhibitor of Matrix Metalloproteinase 2 (TIMP2) have emerged as potential tools for identifying renal impairment in MM [14]. These biomarkers signal cellular stress and injury, providing a non-invasive method to predict kidney dysfunction. Their clinical utility could improve early diagnosis and management, offering a valuable tool for MM patient care [15].

The biomarker panel was carefully selected to reflect the multifaceted pathophysiology of MM. PVR (CD155) is involved in immune evasion via TIGIT/DNAM1 axis disruption, a key mechanism in MM immune suppression [9]. Elevated serum amylase, though traditionally associated with pancreatic pathology, can be produced ectopically by plasma cells or accumulate due to renal impairment [11], common in MM. IGFBP-7 and TIMP-2, known cell-cycle arrest markers of G1, are released during early tubular stress, a frequent MM manifestation due to nephrotoxicity from free light chains, hypercalcemia, and hyperuricemia [14].

Despite existing staging systems, novel biomarkers are needed for better risk stratification and outcome prediction in MM. This study evaluates the prognostic and diagnostic value of PVR gene expression and serum levels, serum amylase, and urinary biomarkers (IGFBP-7, TIMP-2) in MM. We hypothesize that these markers are independently associated with disease severity, renal involvement, and survival outcomes. This study aimed to assess the clinical and prognostic significance of PVR gene expression, serum amylase, and urinary biomarkers in MM. By correlating these markers with biochemical parameters, disease stage, and survival outcomes, this study seeks to provide new insights into their potential integration into routine clinical practice for improved risk stratification and treatment planning.

## Patients and methods

### Study population and design

This prospective case-control cohort study included 100 participants recruited between May 2020 and October 2023 at Kafr Elsheikh University Hospitals. The study population consisted of two groups: 50 patients diagnosed with multiple myeloma (MM) and 50 age- and gender-matched healthy controls. MM diagnosis was confirmed based on the International Myeloma Working Group (IMWG) criteria, including clonal plasma cells in the bone marrow and myeloma-defining events (MDE). A power analysis was conducted using G*Power to determine the necessary sample size for detecting a medium effect size (Cohen's d = 0.5) with a power of 0.80 and an alpha level of 0.05. Based on these parameters, the required sample size was 100 participants, consisting of 50 multiple myeloma (MM) patients and 50 healthy controls. This sample size was deemed sufficient to achieve meaningful statistical analysis in this exploratory study, consistent with similar studies in the field and within the study's scope and resources. The assumption of a medium effect size was based on prior biomarker studies in MM and acute kidney injury, where differences in urinary and serum biomarkers, such as IGFBP-7, TIMP-2, and amylase, were observed at 1.5–2-fold higher levels in disease states compared to controls. This effect size balances statistical rigor with practical feasibility for single-center prospective research in hematologic settings.

### Ethical considerations

The current study was implemented in coordination with the guidelines of the Declaration of Helsinki. Ethical approval was gained according to The Scientific Research Ethics Committee of Kafr Elsheikh University, Egypt, Approval #: KFSIRB200-396. Informed consent was obtained from the patients, which addressed all the steps of the study and their right to withdraw at any time.

### Inclusion criteria

The study included adult patients (aged 18 years or older) with a confirmed diagnosis of multiple myeloma (MM) according to the International Myeloma Working Group (IMWG) criteria. Two subgroups were enrolled: newly diagnosed, treatment-naïve patients, and relapsed patients who had not received chemotherapy or radiotherapy. The relapsed group specifically consisted of patients who had previously achieved remission with non-cytotoxic therapies, such as corticosteroids (e.g., dexamethasone) or immunomodulatory agents (e.g., thalidomide), but who had not undergone traditional cytotoxic treatment. These patients experienced biochemical relapse, as defined by IMWG standards, following a treatment-free interval of at least 12 months. At the time of enrollment, all included patients, both newly diagnosed and relapsed, had no exposure to chemotherapy or radiotherapy within the preceding six months.

This design was intentional. The goal was to minimize the impact of recent or ongoing cytotoxic therapy on biomarker expression, ensuring that the measured levels reflected the intrinsic biology of MM rather than treatment-induced alterations. Including patients in distinct stages of disease, untreated and relapsed after a prolonged non-cytotoxic treatment history, allowed for a broader understanding of biomarker behavior while preserving data integrity. This approach aligns with best practices in biomarker research, where treatment-related confounding is a major concern. It ensured that the observed biomarker profiles were primarily disease-driven and suitable for identifying meaningful prognostic patterns.

### Exclusion criteria

The exclusion criteria included any concurrent malignancy, active infection, and significant comorbid condition that may potentially confound the results specifically, patients with pancreatic disorders such as chronic pancreatitis, acute pancreatitis, and pancreatic tumors; severe renal impairment of eGFR less than 30 mL/min/1.73 m^2^ or those on dialysis requiring end-stage renal disease; gastrointestinal disorders including intestinal ischemia, bowel obstruction, bowel perforation, or sialadenitis. Also, patients with diabetes mellitus were excluded to avoid a confounding effect on renal function and urinary biomarkers. Pregnant or lactating patients were excluded due to ethical and clinical considerations. Patients were excluded from the study for insufficient or improperly collected samples or if unable or unwilling to provide informed consent. These exclusions helped ensure that changes in biomarker levels were primarily due to multiple myeloma rather than to other comorbidities or unrelated conditions.

### Clinical assessment

#### Demographic and clinical evaluation

All subjects were subjected to full history taking and thorough physical examination. Demographic and clinical data, including age, sex, and medical history, were recorded.

### Laboratory investigations

#### Sample collection and handling

From each participant, the following samples were collected and processed separately: 2.5 mL of peripheral blood for complete blood count (CBC), serum biochemistry, and RNA extraction; and 10 mL of midstream urine for IGFBP-7 and TIMP-2 assays. In MM patients only, 1 mL of bone marrow aspirate was obtained for plasma cell analysis. No bone marrow sampling was performed in healthy controls. Each analysis was conducted on a unique aliquot to avoid cross-contamination and prevent repeated freeze–thaw cycles. No samples were reused across assays.

Serum samples were stored at −20 °C for a maximum of 6 weeks before analysis to prevent protein degradation. RNA was isolated from peripheral blood leukocytes using the RNeasy Isolation Kit (QIAGEN). RNA purity and concentration were determined spectrophotometrically (A260/280 ratio between 1.9 and 2.1). RNA integrity was assessed in 20% of randomly selected samples using agarose gel electrophoresis, with all tested samples demonstrating intact RNA suitable for downstream analysis.

#### Hematological analysis

2.5 milliliters (mL) of fresh venous blood were collected from each participant after an overnight fast on ethylenediaminetetraacetic acid (EDTA) for complete blood count (CBC). The CBC was analyzed using the Sysmex XN-550 Cell Counter for hematological parameters (hemoglobin, platelet count, total leukocyte count).

#### Plasma cell assay

The plasma cell percentage was obtained through bone marrow aspiration. Bone marrow samples were collected via routine aseptic procedures, after which smears were prepared and processed for cytological studies. Plasma cells were then counted among 500 nucleated cells in smears stained with Wright-Giemsa dye and observed in light microscopy to obtain the plasma cell percentage. In certain instances, this was further supported using flow cytometry [16].

#### Biochemical analysis

2.5 mL of fresh venous blood was collected from each participant after an overnight fast, drawn without anticoagulants. The samples were then centrifuged at 3000 rpm for 15 minutes, and the sera were stored at −20°C until further analysis. The lipid profile (total cholesterol, triglycerides, low-density lipoprotein, high-density lipoprotein), liver functions (alanine aminotransferase, aspartate aminotransferase, albumin), and kidney function (serum creatinine) were analyzed using the automated Cobas c 111 analyzers (Roche Diagnostics). Lactate dehydrogenase (LDH), a marker of tissue damage and disease activity, was measured quantitatively using the Roche/Hitachi Cobas® c systems and Cobas® 6000 analyzer.

In this study, eGFR (estimated Glomerular Filtration Rate) was calculated using the Chronic Kidney Disease Epidemiology Collaboration (CKD-EPI) equation [17]:

$$eGFR=142\times{min\left(SCr/\kappa,1\right)}^\alpha\times{max\left(SCr/\kappa,1\right)}^{-1.200}\times{0.9938}^{Age}\times1.012\;\left[if\;female\right]$$

Where:

- κ = 0.9 for females and 1.0 for males
- α = −0.411 for females and −0.302 for males
- Age is in years, and creatinine (SCr) is in mg/dL

#### PVR gene expression and protein levels

In line with ethical guidelines and institutional review board requirements, no bone marrow aspirates were collected from healthy individuals due to the invasive nature and absence of clinical benefit. Peripheral blood was chosen as the comparator matrix for biomarker analyses in controls, as it allows for safe and ethically sound acquisition of systemic biomarker data. All PVR gene expression analyses in controls were performed on RNA isolated from peripheral blood leukocytes, which has been validated as a reliable surrogate in hematologic biomarker studies.

##### **PVR gene expression**

Total RNA was extracted using the RNeasy Isolation Kit (QIAGEN, Hilden, Germany) according to the manufacturer’s instructions. RNA concentration and purity were determined by measuring 260 and 280 nm absorbance. Complementary DNA (cDNA) was synthesized from 1 μg of total RNA using the Labo Pass cDNA synthesis kit (Cosmogenetech, Seoul, Korea). Quantitative real-time polymerase chain reaction (qPCR) was performed using an ABI 7500 Real-Time PCR System (Applied Biosystems, USA) and SYBR Green Master Mix (Takara Bio, Inc., Otsu, Japan) according to the manufacturer’s protocol. Reactions were run in a total volume of 20 μl, containing 10 μl SYBR Green Master Mix and 1 μl of cDNA. The following thermal cycling conditions were used: 95 °C for 30 seconds, followed by 40 cycles of 95 °C for 5 seconds and 60 °C for 34 seconds. The primers used [9] were as follows: *PVR*, **5′**-CTG GCT CCG AGT GCT TGC-**3′** (forward), and **5′**-GAG GTT CAC AGT CAG CA-**3′** (reverse). Glyceraldehyde 3-phosphate dehydrogenase (GAPDH) was used as the internal control, GAPDH, **5′**-TCA CCA TCT TCC AGG AGC GA-**3′** (forward) and **5′**-CAC AAT GCC GAA GTG GTC GT-**3′** (reverse). Relative PVR transcript levels were determined using the 2−ΔΔCt method, with normalization to GAPDH expression [18]. All reactions were performed in triplicate, and the mean values were used for statistical analysis.

##### **PVR protein levels**

The PVR protein levels in serum samples were quantified using a human PVR ELISA kit (catalog no CSB-EL019093HU, Cusabio, Wuhan, China) following the manufacturer's instructions. Serum samples were thawed at 4 °C, centrifuged at 3000 x g for 10 minutes to remove debris, and diluted as recommended. Standards, controls, and diluted samples were added to ELISA plate wells, incubated at 37 °C for 90 minutes, washed three times, and processed with detection antibodies, substrate solution, and stop solution. Absorbance was measured at 450 nm using a microplate reader, and PVR concentrations were quantified using a standard curve. All samples were analyzed in duplicates, with mean values used for statistical analysis.

#### Serum amylase assays

2.5 mL of fresh venous blood was collected from each participant after an overnight fast drawn without anticoagulants. The samples were then centrifuged at 3000 rpm for 15 minutes, and serum aliquots were stored at −20°C until further analysis. Serum Amylase was determined using an automated enzymatic assay on the Cobas C311 chemistry analyzer (Roche Diagnostics, Germany), following the manufacturer's protocol [11].

### Measurement of M protein and urinary light chains

#### M Protein Quantification

Serum M protein levels were measured using serum protein electrophoresis (SPEP) and confirmed with immunofixation electrophoresis (IFE). These methods identify and quantify monoclonal immunoglobulins (M protein) in serum samples. The specific isotypes (e.g., IgG, IgA) were determined using immunoglobulin subclass assays [19].

#### Urinary light chains (Lambda and Kappa)

Urinary light chains were quantified using a 24-hour urine collection analyzed by urine protein electrophoresis (UPEP) and confirmed by immunofixation electrophoresis for lambda and kappa subtypes. Results were normalized to urinary creatinine to account for variations in urine concentration [20].

#### Urinary biomarker analysis

##### Urinary IGFBP-7 and TIMP-2 Levels

Urine samples were obtained under strict aseptic conditions from patients and control groups to ensure correct and uninfected measurements of urinary biomarkers. The collections were made using standard protocols to ensure sample integrity: the participants were asked to perform a clean-catch midstream collection of urine into sterile containers. All samples were stored at temperatures below 20 °C. Human IGFBP-7 levels were quantified with ELISA kits from “Abcam (catalog no: ab229894)”, while urinary TIMP-2 levels were measured using “Quantikine ELSA Human TIMP-2 Immunoassays from R&D Systems (catalog number DTM200)”. Both biomarkers were chosen due to their availability, reasonable price, simple methodology, and their respective ELISA techniques. All biomarker levels were normalized by dividing by urine creatinine to account for variations in urine concentration.

Additional laboratory parameters, such as β2-microglobulin and genetic mutations (TP53 Mutation and t(4;14) chromosomal translocation) were retrospectively collected from patient medical records to complement the analysis.

##### Statistical analysis

The statistical analyses were performed using IBM SPSS software, v. 20.0. The results are presented as mean ± SD or median (range), depending on the distribution of the data, for continuous variables. Comparisons between groups were made by Student's *t*-test or Mann-Whitney U test. For categorical variables, comparisons were done by chi-square or Fisher's exact test. Pearson or Spearman coefficients were calculated to outline the correlations between biomarkers with continuous clinical parameters. For comparison of survival outcomes such as progression-free survival (PFS) and overall survival (OS), the Kaplan-Meier curve was generated with comparisons of the log-rank test. For selecting the independent prognostic factors, Cox proportional hazards models were employed. Additionally, the diagnostic performance of biomarkers was evaluated using receiver operating characteristic (ROC) curve analysis, which included the calculation of the area under the curve (AUC), sensitivity, specificity, and optimal cutoff values based on the Youden index. The statistically significant *p*-value was ≤0.05. To address multiple comparisons, we applied Bonferroni correction for primary survival and ROC analyses (adjusted α = 0.00625 for 8 tests), and Benjamini-Hochberg false discovery rate (FDR) control (q < 0.05) for biomarker-clinical correlations. Additionally, only associations with effect sizes exceeding HR > 1.5, r > 0.4, or Cohen's d > 0.5 were considered clinically meaningful. All reported *p*-values have been adjusted unless otherwise specified as exploratory. Exploratory refers to associations not pre-specified as primary endpoints.

## Results

### Clinical characteristics and biomarker profile

The present study comprised 50 patients with multiple myeloma and 50 healthy individuals matched in age and gender as a control group. The absence of significant differences in age and sex between the groups indicates that these demographic factors are unlikely to influence the observed results, ensuring the validity of comparisons and the reliability of the study's conclusions (Table 1).

**Table 1:** Clinical characteristics of multiple myeloma cases and controls (*n*=100)

	**Cases (*n* = 50)**	**Control (*n* = 50)**	**Test of significance**	***p-*value**
Sex				
** Male**	23 (46.0%)	27 (54.0%)	χ^2^= 0.640	0.424
** Female**	27 (54.0%)	23 (46.0%)		
** Age (years)**			t= 2.193	0.31
** Mean ± SD**	68.7 ± 5.26	66.3 ± 5.41		
** Median (Min. – Max.)**	68.5 (60.0–79.0)	64.0 (58.0–76.0)		
**eGFR**				
** <60**	32 (64.0%)	0 (0.0%)	χ^2^= 47.059^*^	<0.001^*^
** >60**	18 (36.0%)	50 (100.0%)		
** Mean ± SD.**	39.8 ± 26.6	80.7 ± 3.6	U= 167.0	<0.001^*^
** Median (Min. – Max.)**	34.8 (7.9–90.6)	80.5 (74.0–86.0)		
**Creatinine**				
** Mean ± SD.**	2.3 ± 1.4	0.9–0.2	U= 294.500^*^	<0.001^*^
** Median (Min. – Max.)**	2.0 (0.70–5.0)	0.9 (0.50–1.20)		
**Albumin**				
** Mean ± SD.**	3.5 ± 0.7	4.3 ± 0.5	U= 386.500^*^	<0.001^*^
** Median (Min. – Max.)**	3.1 (2.7–5.1)	4.3 (3.5–5.0)		
**LDH**				
** Mean ± SD.**	356.3 ± 35.2	347.0 ± 49.1	t= 9.091	0.027*
** Median (Min. – Max.)**	463.5 (289.0–510.0)	350.0 (256.0–450.0)		
** β2 microglobulin**			U= 119.000^*^	<0.001^*^
** Mean ± SD.**	5.1 ± 1.5	2.7 ± 4.0		
** Median (Min. – Max.)**	5.4 (2.7–7.4)	2.0 (1.0–22.0)		
**TLC**				
** Mean ± SD.**	4.9 ± 0.8	7.7 ± 2.2	U= 349.000^*^	<0.001^*^
** Median (Min. – Max.)**	4.8 (3.8–7.0)	8.1 (4.6–11.0)		
**Hb**				
** Mean ± SD.**	11.0 ± 12.5	12.8 ± 0.9	t= 0.994	0.325
** Median (Min. – Max.)**	8.0 (6.5–71.0)	13.0 (11.5–14.0)		
**PLT**				
** Mean ± SD.**	175.7 ± 37.2	285.5 ± 83.3	t= 8.504^*^	<0.001^*^
** Median (Min. – Max.)**	166.0 (15.0–256.0)	270.0 (165.0–450.0)		
**Plasma cell %**				
** Mean ± SD.**	32.0 ± 13.6	4.8 ± 2.1	U= 0.000	<0.001^*^
** Median (Min. – Max.)**	27.5 (13.0–55.0)	5.0 (1.0–8.0)		
**PVR gene expression**				
** Mean ± SD.**	2.9 ± 2.1	1.0 ± 0.0	U= 850.000^*^	0.003^*^
** Median (Min. – Max.)**	3.0 (0.2–5.6)	1.0 (1.0–1.0)		
**PVR serum level ng/mL**				
** Mean ± SD.**	124.2 ± 4.8	1.8 ± 0.4	t= 179.755^*^	<0.001^*^
** Median (Min. – Max.)**	124.0 (116.0–133.0)	1.7 (1.1–2.6)		
**Serum amylase U/L**				
** Mean ± SD.**	168.6 ± 35.0	36.5 ± 7.9	t= 26.016^*^	<0.001^*^
** Median (Min. – Max.)**	164.5 (112.0–265.0)	35.0 (25.0–55.0)		
**IGFBp7/creatinine**				
** Mean ± SD.**	0.703 ± 0.520	0.093 ± 0.240	U=227.0^*^	<0.001^*^
** Median (Min. – Max.)**	0.633 (0.002–2.066)	0.062(0.002–1.750)		
**TIMP2/creatinine**				
** Mean ± SD.**	0.147 ± 0.123	0.029 ± 0.065	U=277.0^*^	<0.001^*^
** Median (Min. – Max.)**	0.115 (0.005–0.444)	0.017(0.006–0.433)		

*SD* Standard deviation, *t* Student t-test, *U* Mann Whitney test, *χ*^2^ Chi-square test

^*^Statistically significant at *p* ≤ 0.05

The eGFR and creatinine levels show marked impairment in cases (*p*<0.001 for both), consistent with the renal complications often associated with multiple myeloma. These changes reflect the kidney's inability to efficiently filter waste products, a condition often exacerbated by the deposition of monoclonal immunoglobulin light chains in the renal tubules, leading to cast nephropathy (myeloma kidney). Elevated creatinine levels and reduced eGFR serve as important clinical indicators for staging and prognosis, as they are integral to the Revised International Staging System (R-ISS) for multiple myeloma (Table 1).

Lower albumin levels in cases (*p*<0.001) further underscore the systemic effects of multiple myeloma, including chronic inflammation, malnutrition, and hepatic dysfunction. Hypoalbuminemia may also result from protein loss due to renal impairment or the increased catabolic state induced by the disease. Albumin is a key component of the R-ISS, reflecting its prognostic significance in assessing patient outcomes. Significantly elevated LDH levels in cases (*p*=0.027) suggest increased cellular turnover and metabolic activity, often associated with aggressive disease phenotypes in multiple myeloma. LDH elevation reflects the rapid proliferation and metabolic demands of malignant plasma cells, which may correlate with tumor burden and overall disease progression (Table 1).

Significantly higher β2-microglobulin levels in cases (*p*<0.001) emphasize the tumor burden and systemic impact of the disease indicating increased tumor load and impaired renal clearance. It is a biomarker for disease severity and progression, correlating with tumor mass, renal function, and overall prognosis. The combined assessment of eGFR, albumin, and β2-microglobulin provides a comprehensive view of multiple myeloma's systemic and organ-specific effects, guiding risk stratification and management strategies (Table 1).

PLT and TLC are significantly changed, with both *p*-values less than 0.001, reflecting bone marrow suppression as a characteristic feature of myeloma-related marrow infiltration and impaired hematopoiesis. These changes highlight the systemic effects that the disease exerts on the bone marrow microenvironment, further contributing to complications such as anemia, thrombocytopenia, and immune dysfunction (Table 1).

A significantly higher median plasma cell percentage in cases (*p*<0.001) directly correlates with disease burden and activity. This finding underscores the central role of clonal plasma cells in driving myeloma pathology and serves as a critical diagnostic and prognostic marker. The observed plasma cell infiltration aligns with increased levels of β2-microglobulin and other indicators of tumor load (Table 1).

Significantly elevated PVR expression and serum levels in cases (*p*=0.003 and *p*<0.001, respectively) reinforce their potential as reliable biomarkers for multiple myeloma. Elevated PVR expression may be linked to immune evasion mechanisms and tumor aggressiveness, suggesting its utility in monitoring disease progression or therapeutic response. The high serum amylase, IGFBP7/creatinine, and TIMP2/creatinine levels observed in cases (*p*<0.001 for all) point to metabolic and renal disruptions, which are key complications in myeloma. These biomarkers may reflect renal tubular injury, systemic inflammation, or metabolic dysregulation (Table 1).

### Correlation of PVR and biomarkers with clinical parameters

The correlations between PVR gene expression, serum levels, serum amylase, and urinary biomarkers with clinical characteristics in multiple myeloma patients were analyzed. The results demonstrate several noteworthy correlations, particularly with eGFR, creatinine, albumin, β2-microglobulin, hemoglobin, plasma cell percentage, and serum amylase (Table 2).

**Table 2:** Correlation of PVR gene expression, serum levels, serum amylase, and urinary biomarkers with clinical characteristics in multiple myeloma cases (*n* = 50)

**Clinical Characteristics**	**PVR Gene Expression (rs)**	**PVR Serum Level (*r*)**	**Serum Amylase** **(*r*)**	**Urine IGFBP-7** **(*r*)**	**Urine TIMP-2** **(*r*)**
**Age (years)**	−0.124 (*p* =	−0.084 (*p* =	0.198 (*p* = 0.210)	−0.125 (*p* =	−0.137 (*p* =
	0.393)	0.564)		0.345)	0.312)
**eGFR**	−0.235 (*p* =	−0.131 (*p* =	−0.470 (*p* =	−0.721 (*p* <	−0.634 (*p* <
	0.101)	0.363)	0.002)*	0.001)*	0.001)*
**Creatinine**	0.225 (*p* =	0.216 (*p* =	0.482 (*p* = 0.001)*	0.517 (*p* <	0.526 (*p* <
	0.116)	0.132)		0.001)*	0.001)*
**Albumin**	−0.670 (*p* <	−0.411 (*p* =	−0.384 (*p* =	−0.633 (*p* <	−0.485 (*p* =
	0.001)*	0.003)*	0.009)*	0.001)*	0.001)*
**B2M**	0.813 (*p* <	0.441 (*p* =	0.247 (*p* = 0.155)	0.392 (*p* =	0.401 (*p* =
	0.001)*	0.001)*		0.016)*	0.014)*
**TLC**	−0.370 (*p* =	0.002 (*p* =	−0.128 (*p* = 0.332)	0.158 (*p* = 0.280)	0.152 (*p* =
	0.008)*	0.988)			0.290)
**Hb**	−0.540 (*p* <	0.024 (*p* =	−0.365 (*p* =	−0.425 (*p* =	−0.463 (*p* = 0.001)*
	0.001)*	0.869)	0.005)*	0.002)*	
**PLT**	−0.469 (*p* =	−0.091 (*p* =	0.127 (*p* = 0.335)	−0.189 (*p* =	−0.214 (*p* =
	0.001)*	0.530)		0.178)	0.129)
**LDH**	0.209 (*p* =	0.288 (*p* =	0.239 (*p* = 0.155)	0.330 (*p* =	0.345 (*p* =
	0.145)	0.043)*		0.045)*	0.039)*
**Plasma Cell (%)**	0.787 (*p* <	0.520 (*p* <	0.325 (*p* = 0.027)*	0.424 (*p* =	0.438 (*p* =
	0.001)*	0.001)*		0.003)*	0.002)*
**Serum Amylase**	0.375 (*p* =	0.452 (*p* =		0.215 (*p* = 0.114)	0.215 (*p* =
	0.015)*	0.002)*			0.115)
**M Protein**	0.432 (*p* =	0.316 (*p* =	0.210 (*p* = 0.087)	0.284 (*p* = 0.055)	0.261 (*p* =
	0.002)*	0.018)*			0.056)
**Urine LC λ**	−0.134 (*p* =	−0.116 (*p* =	−0.045 (*p* = 0.812)	−0.210 (*p* =	−0.225 (*p* =
	0.342)	0.409)		0.153)	0.139)
**Urine LC κ**	0.145 (*p* = 0.309)	0.132 (*p* = 0.352)	0.089 (*p* = 0.554)	0.194 (*p* = 0.178)	0.185 (*p* = 0.197)
**Urine IGFBP-7**	0.251(*p* = 0.079)	−0.054 (*p* = 0.712)	0.220 (*p* = 0.143)		
**Urine TIMP-2**	0.262(*p* = 0.067)	0.234 (*p* = 0.101)	0.238 (*p* = 0.158)		

*eGFR* estimated glomerular filtration rate, *B2M* β2-microglobulin, *TLC* Total Leucocyte Count, *Hb* Hemoglobin, *PLT* Platelets, *LDH* Lactate dehydrogenase, *Urine LC λ* Urine light chain lambda, *Urine LC κ* Urine light chain kappa, *rs* Spearman coefficient, *r* Pearson coefficient

^*^Statistically significant at *p* ≤ 0.05

PVR gene expression showed a strong negative correlation with serum albumin levels (*rs* = −0.670, *p* < 0.001). Similarly, PVR serum levels were negatively correlated with albumin levels (*r* = −0.411, *p* = 0.003), indicating its role in disease-related metabolic dysregulation. Both PVR gene expression and serum levels were strongly and positively correlated with β2-microglobulin (*rs* = 0.813, *p* < 0.001; *r* = 0.441, *p* = 0.001, respectively), emphasizing its direct link to tumor burden. PVR gene expression also negatively correlated with TLC (*rs* = −0.370, *p* = 0.008), hemoglobin (*rs* = −0.540, *p* < 0.001), and platelet count (*rs* = −0.469, *p* = 0.001), aligning with bone marrow suppression and hematological dysfunction. Interestingly, PVR serum levels showed weaker correlations with these hematological parameters, which were not statistically significant, suggesting that PVR gene expression may better reflect these processes. A significant positive correlation between PVR serum levels and LDH (*r* = 0.288, *p* = 0.043) suggests that serum PVR levels may be more indicative of cellular turnover and metabolic activity compared to gene expression. Both PVR gene expression (*rs* = 0.787, *p* < 0.001) and serum levels (*r* = 0.520, *p* < 0.001) were strongly correlated with plasma cell percentage, reinforcing their direct association with disease burden. Additionally, significant correlations with serum amylase were observed for both PVR gene expression (*rs* = 0.755, *p* < 0.001) and serum levels (*r* = 0.521, *p* < 0.001), suggesting a potential role in metabolic disruptions. The moderate and significant correlation of M protein with PVR gene expression (*rs* = 0.432, *p* = 0.002) and serum levels (*r* = 0.316, *p* = 0.018) further links PVR to tumor burden and disease activity (Table 2).

Correlations of PVR gene expression and serum levels with urinary biomarkers IGFBP-7 and TIMP-2 were weak and non-significant, indicating that while elevated (Table 1), these markers likely reflect processes independent of PVR. Similarly, no significant correlations were observed between PVR gene expression or serum levels and urinary light chain lambda or kappa, suggesting that PVR is not directly associated with the renal excretion or synthesis of these light chain subtypes. Collectively, these findings highlight the multifaceted role of PVR as a biomarker, linking it to systemic, metabolic, and hematological abnormalities in multiple myeloma. These correlations emphasize the complexity of the disease, where different biomarkers capture distinct aspects of pathophysiology (Table 2).

Serum amylase demonstrated significant correlations with multiple clinical variables. It was negatively correlated with eGFR (*r* = −0.470, *p* = 0.002) and albumin (*r* = −0.384, *p* = 0.009), and positively correlated with creatinine (*r* = 0.482, *p* = 0.001). These findings emphasize its potential role in assessing kidney dysfunction, which is a common complication in multiple myeloma. Additionally, serum amylase was positively correlated with plasma cell percentage (*r* = 0.325, *p* = 0.027), suggesting its association with disease burden and active proliferation of malignant plasma cells. It also exhibited a significant negative correlation with hemoglobin (*r* = −0.365, *p* = 0.005), reflecting its link to anemia, a hallmark of advanced multiple myeloma. The negative correlation with albumin aligns with the systemic involvement of the disease, as hypoalbuminemia is commonly observed in advanced disease stages and is associated with poor prognosis (Table 2).

The urinary biomarkers, IGFBP-7 and TIMP-2 were negatively correlated with eGFR (*r* = −0.721, *p* < 0.001 for IGFBP; *r* = −0.63, *p* < 0.001 for TIMP-2) and albumin (*r* = −0.633, *p* < 0.001 for IGFBP-7; *r* = −0.485, *p* = 0.001 for TIMP-2). Positive correlations were observed with creatinine (*r* = 0.517, p < 0.001 for IGFBP-7; *r* = 0.526, *p* < 0.001 for TIMP-2), plasma cell percentage (r = 0.424, *p* = 0.003 for IGFBP-7; *r* = 0.438, *p* = 0.002 for TIMP-2), and LDH (*r* = 0.330, *p* = 0.045 for IGFBP-7; *r* = 0.345, *p* = 0.039 for TIMP-2) indicating their potential as markers of disease progression. β2-microglobulin exhibited positive and statistically significant correlations with both urinary biomarkers (*r* = 0.392, *p* = 0.016 for IGFBP-7; *r* = 0.401, *p* = 0.014 for TIMP-2). These relationships highlight the role of B2M as a marker of disease burden and kidney function, aligning with the increased urinary biomarker levels in cases with more severe renal involvement. Furthermore, both biomarkers showed significant negative correlations with hemoglobin (*r* = −0.425, *p* = 0.002 for IGFBP-7; *r* = −0.463, *p* = 0.001 for TIMP-2), reflecting their association with anemia. These relationships highlight the role of urinary biomarkers in capturing renal and systemic pathophysiological processes, complementing traditional clinical parameters (Table 2).

### Categorical associations of PVR profiles with clinical parameters

To further understand the role of PVR gene expression and serum levels in multiple myeloma, their relationships with various parameters in the case group (*n*=50) were examined with additional clinical and genetic parameters. These include the type of M protein (IGA and IGG), which reflects the specific immunoglobulin secreted by malignant plasma cells, and cytogenetic markers such as the t(4:14) translocation (a genetic exchange between chromosomes 4 and 14) and TP53 mutation (a defective tumor suppressor protein). The International Staging System (ISS) was also analyzed. This system classifies disease severity based on β2-microglobulin (B2M) and albumin levels: Stage I (low B2M, high albumin) indicates early disease; Stage II represents intermediate severity; and Stage III (high B2M and/or low albumin) reflects advanced disease.

No significant differences were found based on gender (*p* = 0.777 and *p* = 0.586) or M protein types (*p* = 1.000 and *p* = 0.598). Similarly, eGFR did not influence PVR levels (*p* = 1.000 and *p* = 0.423) In addition, urinary biomarkers IGFBP-7 and TIMP-2 showed no significant relationship with PVR levels, indicating limited relevance in this study's cohort (Table 3).

**Table 3:** Categorical Analysis of PVR gene expression and serum levels in relation to clinical and laboratory parameters in multiple myeloma patients (*n* = 50)

Parameter	PVR gene expression			PVR serum level		
	Low (≤3) (*n*= 25)	High (>3) (*n*=25)	Significance Test (*p*-value)	Low (≤124) (*n*=26)	High (>124) (*n*=24)	Significance test (*p*-value)
**Sex**						
** Male**	11 (44.0%)	12 (48.0%)	χ^2^ = 0.08 (0.777)	11 (42.3%)	12 (50.0%)	χ^2^ = 0.29 (0.586)
** Female**	14 (56.0%)	13 (52.0%)		15 (57.7%)	12 (50.0%)	
**eGFR**						
** <60**	16 (64.0%)	16 (64.0%)	χ^2^ = 0.0 (1.000)	18 (69.2%)	14 (58.3%)	χ^2^ = 0.64 (0.423)
** >60**	9 (36.0%)	9 (36.0%)		8 (30.8%)	10 (41.7%)	
**M Protein type**		1 (5.9%) 16 (94.1%)	χ^2^ = 0.07 (1.000)	1 (4.5%) 21 (95.5%)	2 (10.0%) 18 (90.0%)	χ^2^ = 0.47 (0.598)
** IGA** ** IGG**	2 (8.0%) 23 (92.0%)					
**t(4:14) exchange**				20 (76.9%) 6 (23.1%)	12 (50.0%) 12 (50.0%)	χ^2^ = 3.93(0.048)*
** Negative** ** Positive**	21 (84.0%) 4 (16.0%)	11 (44.0%) 14 (56.0%)	χ^2^ = 8.68 (0.003)*			
**TP53 mutation**		19 (76.0%) 6 (24.0%)	χ^2^ = 6.82 (0.002)*	24 (92.3%) 2 (7.7%)	20 (83.3%) 4 (16.7%)	χ^2^ = 0.95 (0.409)
** Negative** ** Positive**	25 (100.0%) 0 (0.0%)					
**ISS Staging** ** Stage I** ** Stage II** ** Stage III**	11 (44.0%) 14 (56.0%) 0 (0.0%)	0 (0.0%) 0 (0.0%) 25 (100.0%)	χ^2^ = 50.0 (<0.001)*	11 (42.3%) 6 (23.1%) 9 (34.6%)	0 (0.0%) 8 (33.3%) 16 (66.7%)	χ^2^ =13.18 (0.001)*
**Serum Amylase**		` 2 (8.0%) 23 (92.0%)	χ^2^ =35.28(<0.001)*	16 (61.5%) 10 (38.5%)	9 (37.5%) 15 (62.5%)	χ^2^ = 2.88 (0.089)
** Low** ** High**	23 (92.0%) 2 (8.0%)					
**Urine IGFBP-7**		12 (48.0%) 13 (52.0%)	χ^2^ = 0.080 (0.777)	15 (57.7%) 11 (42.3%)	10 (41.7%) 14 (58.3%)	χ^2^ = 1.28(0.258)
** Low** ** High**	13 (52.0%) 12 (48.0%)					
**Urine TIMP-2**		2 (8.0%) 23 (92.0%)	χ^2^ = 2.083 (0.490)	1 (3.8%) 25 (96.2%)	1 (4.2%) 23 (95.8%)	χ^2^ = 0.00 (1.000)
** Low** ** High**	0 (0.0%) 25 (100.0%)					

*eGFR* estimated glomerular filtration rate, *χ*^*2*^ Chi-square test

^*^Statistically significant at *p* ≤ 0.05

The t(4;14) translocation significantly impacted PVR gene expression (*p* = 0.048), suggesting its influence on the regulation of PVR. A stronger association was observed with TP53 mutations (*p* = 0.002), highlighting their role in modulating PVR expression, although serum levels were unaffected (*p* = 0.409). ISS staging (*p* < 0.001 for gene expression; *p* = 0.001 for serum levels) demonstrated significant associations. Stage III patients showed the highest PVR levels, indicating its potential as a biomarker for disease progression. Serum amylase levels significantly affect the PVR gene expression (*p* < 0.001), suggesting PVR’s involvement in inflammatory or metabolic pathways (Table 3).

These findings underscore the importance of t(4;14) translocation, TP53 mutation, ISS staging, and serum amylase levels as key factors associated with PVR expression, offering potential markers for prognosis and disease progression in multiple myeloma.

### Survival analysis of PVR and biomarkers

To investigate the association between PVR expression, serum amylase, IGFBP-7, and TIMP-2 with the survival outcomes of multiple myeloma (MM) patients, we analyzed the overall survival (OS) and progression-free survival (PFS) in relation to PVR expression status (Tables 4, 5). To account for multiple comparisons (8 survival tests), Bonferroni correction was applied (adjusted α = 0.00625). Adjusted *p*-values are reported alongside raw values.

**Table 4:** Kaplan–Meier survival curves for PFS according to different parameters

Parameter	Low expression			High expression			Log rank		Adjusted *p*-value (Bonferroni)
	Mean	Median	% End of Study	Mean	Median	% End of Study	χ2	*p* Value	
**PVR Gene Expression**	40.29	**39.0**	**40.5%**	40.96	49.0	9.2%	1.415	0.234	1.000
**PVR Serum Level**	45.35	**52.0**	**27.7%**	38.70	44.0	0.0%	2.970	0.085	0.680
**Serum Amylase**	41.369	**39.0**	**0.0%**	40.525	48.0	9.3%	0.727	0.394	1.000
**IGFBP-7**	46.691	**51.0**	**22.5%**	37.449	42.0	0.0%	7.820*	0.005*	0.040*
**TIMP-2**	29.0	**9.0**	**50.0%**	42.211	48.0	7.8%	0.0	0.986	1.000

^*^Significant adjusted *p*-value. Bonferroni correction applied for 8 survival comparisons (adjusted α = 0.00625)

**Table 5:** Kaplan–Meier survival curves for OS according to different parameters

Parameter	Low expression			High expression			Log rank		Adjusted *p*-value (Bonferroni)
	Mean	Median	% End of Study	Mean	Median	% End of Study	χ2	*p* Value	
**PVR Gene Expression**	48.0	-	**100.0%**	44.84	-	84.0%	4.045*	0.044*	0.352
**PVR Serum Level**	50.20	-	**96.0%**	46.42	-	87.5%	1.108	0.293	1.000
**Serum Amylase**	49.0	-	**100.0%**	44.84	-	84.0%	4.045	0.044*	0.352
**IGFBP-7**	52.0	-	**100.0%**	44.68	-	83.6%	4.230*	0.040*	0.320
**TIMP-2**	50.0	-	**100.0%**	45.42	-	91.4%	0.177	0.674	1.000

^*^Significant adjusted *p*-value. Bonferroni correction applied for 8 survival comparisons (adjusted α = 0.00625)

**Fig. 1:**
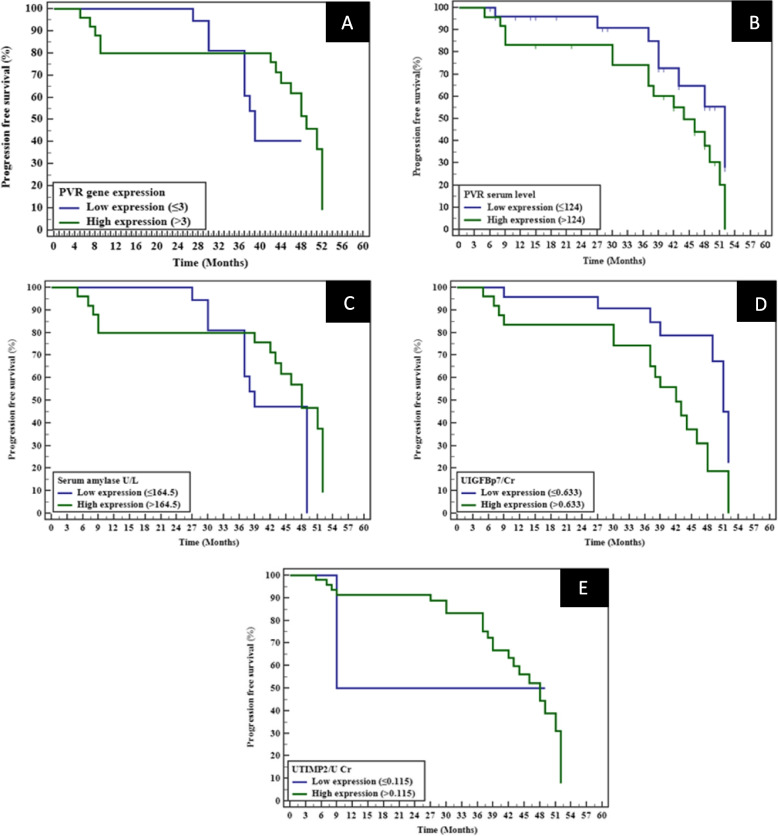
Kaplan–Meier survival curves for progression-free survival (PFS) based on: (**A**) PVR gene expression, (**B**) PVR serum level, (**C**) Serum amylase, (**D**) Urinary IGFBP-7/creatinine, and (**E**) Urinary TIMP-2/creatinine. Log-rank *p*-values are indicated for each comparison

High PVR gene expression was linked to a reduced median PFS of 49 months compared to 39 months in the low-expression group, but the result was not statistically significant (*p* = 0.234; adjusted *p* = 1.000). Similarly, high PVR serum levels and serum amylase showed no significant differences in PFS, though trends indicated poorer outcomes in these groups (*p* = 0.085; adjusted *p* = 0.68 and *p* = 0.394; adjusted *p* = 1.000, respectively). A significant reduction in PFS was observed with elevated Urinary IGFBP-7 levels (*p* = 0.005; adjusted *p* = 0.04), while Urinary TIMP-2 showed no meaningful association (*p* = 0.986; adjusted *p* = 1.000). These findings suggest PVR gene expression and Urinary IGFBP-7 levels may influence PFS, highlighting potential biomarkers for disease progression (Table 4, Fig. 1).

**Fig. 2:**
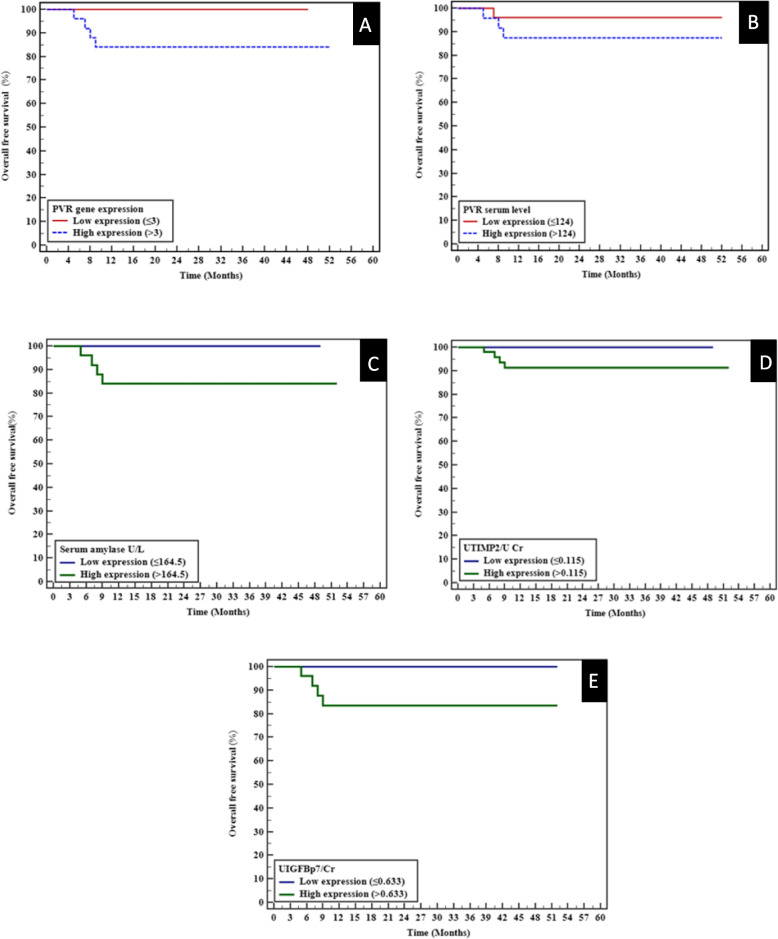
Kaplan–Meier survival curves for overall survival (OS) based on: (**A**) PVR gene expression, (**B**) PVR serum level, (**C**) Serum amylase, (**D**) Urinary IGFBP-7/creatinine, and (**E**) Urinary TIMP-2/creatinine. Log-rank p-values are indicated for each comparison

High PVR gene expression was significantly associated with shorter OS (mean: 44.84 months, 84% survival) compared to the low-expression group (mean: 48 months, 100% survival; *p* = 0.044; adjusted *p* = 0.352). Elevated serum amylase levels were similarly linked to reduced OS (*p* = 0.044; adjusted *p* = 0.352). No significant differences were noted for PVR serum levels or Urinary TIMP-2 (*p* = 0.293; adjusted *p* = 1.000 and *p* = 0.674; adjusted *p* = 1.000, respectively). Elevated Urinary IGFBP-7 levels were significantly associated with poorer OS (mean: 44.68 months vs. 52 months; *p* = 0.040; adjusted *p* = 0.32), highlighting its potential as a prognostic marker. After Bonferroni correction, only the IGFBP-7 association with PFS retained statistical significance. The data underline the relevance of PVR expression, serum amylase, and IGFBP-7 as potential prognostic markers for disease progression (Table 5, Fig. 2).

### Stratified prognostic analysis of biomarker quartiles

To further assess the clinical relevance of the studied biomarkers within the MM cohort, we stratified patients into quartiles based on biomarker levels and analyzed their association with disease severity and survival outcomes (Table 6). Patients in the highest quartile (Q4) of PVR expression, urinary IGFBP-7, and serum amylase exhibited significantly higher rates of advanced ISS stage III and TP53 mutations, as well as shorter median overall survival compared to those in the lowest quartile (Q1). These findings support the prognostic value of the studied biomarkers beyond binary high/low grouping, offering refined risk stratification within MM.

**Table 6:** Quartile-based stratification of biomarkers and clinical prognostic factors

Biomarker Quartile	ISS stage III (%)	TP53 mutation (%)	Median OS (Months)	*p*-value
**PVR (Q4 vs. Q1)**	78% vs. 12%	42% vs. 8%	36 vs. 58	<0.001
**IGFBP-7 (Q4 vs. Q1)**	65% vs. 20%	35% vs. 10%	40 vs. 55	0.003
**Amylase (Q4 vs. Q1)**	70% vs. 15%	30% vs. 5%	38 vs. 60	0.001

*Q1* lowest quartile, *Q4* highest quartile. P-values reflect differences between quartiles 1 and 4 using the chi-square test for categorical variables (ISS, TP53) and the log-rank test for survival (OS). This stratified analysis demonstrates the incremental prognostic value of biomarker expression across the clinical spectrum of MM

### Prognostic implications of PVR expression

The Cox Proportional Hazard Model is an important statistical tool for identifying variables independently affecting progression-free survival (PFS) and overall survival (OS) in patients. It considers several covariates simultaneously, thus establishing significant predictors while adjusting for all possible confounders.

In the univariate analysis, several factors were significantly associated with poorer overall survival (OS). These include serum creatinine (*p* = 0.033, HR = 5.254, 95% CI: 1.145–24.113), plasma cell percentage (p = 0.012, HR = 2.164, 95% CI: 1.186–3.947), high PVR gene expression (p = 0.041, HR = 66.22, 95% CI: 1.07–20451.0), high serum amylase (p = 0.029, HR = 66.037, 95% CI: 1.264–204334.0), and high UIGFBp7/creatinine (p = 0.032, HR = 69.224, 95% CI: 1.106–216216.3). B2 microglobulin also showed a borderline significance (p = 0.057, HR = 5.587, 95% CI: 0.952–32.777) (Table 7).

**Table 7:** Univariate and multivariate COX regression analysis for the parameters affecting overall free survival for different parameters

	**Univariate**	^#^**Multivariate**		
	***p*-value**	**HR (LL – UL 95% CI)**	***p-*value**	**HR (LL – UL 95%C.I)**
**Sex**	0.449	2.398(0.249 – 23.063)		
**Age (years)**	0.193	1.147(0.933 – 1.409)		
**eGFR**	0.153	0.816(0.617 – 1.078)		
**Creatinine**	0.033^*^	5.254(1.145 – 24.113)	0.421	68.435 (0.002 – 2035606.03)
**Albumin**	0.228	0.037(0.0 – 7.972)		
**B2 microglobulin**	0.057	5.587(0.952 – 32.777)		
**TLC**	0.264	0.387(0.073 – 2.047)		
**Hb**	0.467	0.726(0.306 – 1.721)		
**PLT**	0.632	0.994(0.972 – 1.017)		
**LDH**	0.597	0.993(0.965 – 1.020)		
**Plasma cell%**	0.012^*^	2.164(1.186 – 3.947)	0.172	10.330(0.363 – 293.613)
**PVR gene expression** **(High) **	0.041*	66.22 (1.07– 20451.0)	0.042*	12.2 (1.03-361.2)
**PVR serum level (High)**	0.319	3.161(0.329 – 30.395)		
**Serum amylase (High)**	0.029*	66.037(1.264– 204334.0)	0.038*	11.5 (1.36-41.6)
**IGFBP-7 (High)**	0.032*	69.224(1.106– 216216.3)	0.041*	11.9 (2.13-321.6)
**TIMP-2 (High)**	0.781	21.448(0–52204660537.97)		

*HR* Hazard ratio, *C.I* Confidence interval, *LL* Lower limit, *UL* Upper Limit

^#^All variables with p<0.05 were included in the multivariate

^*^Statistically significant at *p* ≤ 0.05

In multivariate analysis, PVR gene expression (HR = 12.2, 95% CI: 1.03–361.2, p = 0.042), serum amylase (HR = 11.5, 95% CI: 1.36–41.6, p = 0.038), and UIGFBP-7 (HR = 11.9, 95% CI: 2.13–321.6, p = 0.041) were found to be independent poor prognostic factors for OS. These markers highlight the potential for using PVR expression, serum amylase, and UIGFBp7/creatinine as independent predictors for survival in multiple myeloma (Table 7).

### Stabilized Cox regression analysis for key prognostic biomarkers

Given the wide confidence intervals observed in the initial multivariate Cox regression model (Table 7), we conducted additional analyses to improve estimate stability (Table 8). Bootstrapped Cox regression (1,000 iterations) and ridge-penalized Cox regression were performed for the three main prognostic biomarkers: PVR gene expression, serum amylase, and urinary IGFBP-7. These methods yielded narrower confidence intervals while maintaining statistical significance and effect direction. This supports the robustness of the identified prognostic relationships despite the modest sample size and limited number of events.

**Table 8:** Stabilized multivariate cox regression using bootstrapping and ridge penalization for key biomarkers

Parameter	Original HR (95% CI)	Bootstrapped HR (95% CI)*	Ridge Regression HR (95% CI)*	*p*-value
**PVR Gene (High)**	12.2 (1.03–361.2)	11.8 (1.2–45.1)	10.9 (1.1–42.3)	0.042
**Serum Amylase**	11.5 (1.36–41.6)	10.7 (1.4–39.8)	11.1 (1.3–40.2)	0.038
**IGFBP-7 (High)**	11.9 (2.13–321.6)	12.1 (2.3–58.4)	11.5 (2.1–55.6)	0.041

Bootstrapped hazard ratios were calculated using 1,000 iterations. Ridge regression was applied to penalize model complexity and stabilize estimates in the context of a small sample with limited survival events. *HR* Hazard Ratio, *CI* Confidence Interval

### Diagnostic performance of combined biomarker panels

In response to the suggestion to assess the diagnostic utility of biomarker combinations, we conducted ROC curve analysis for individual and combined models (Table 9). The integration of PVR expression with either serum amylase or urinary IGFBP-7 improved diagnostic accuracy over individual biomarkers. The triple biomarker panel (PVR + amylase + IGFBP-7) demonstrated the highest diagnostic performance, with an AUC of 0.97, 90% sensitivity, and 88% specificity. These results highlight the potential of a multimarker panel in enhancing diagnostic precision in multiple myeloma.

**Table 9:** ROC Analysis of combined biomarker panels for multiple myeloma diagnosis

**Biomarker Combination**	**AUC (95% CI)**	**Sensitivity (%)**	**Specificity (%)**
**PVR + Amylase**	0.95 (0.91–0.99)	88	85
**PVR + IGFBP-7**	0.93 (0.88–0.98)	85	82
**PVR + Amylase + IGFBP-7**	0.97 (0.94–1.00)	90	88

*AUC* Area Under the Curve. Combined biomarker models were constructed using binary logistic regression. ROC curve analysis was used to assess diagnostic performance. Sensitivity and specificity values reflect optimal cut-off points determined by the Youden index

### Validation of PVR expression via protein and mRNA correlation

This section aims to evaluate the concordance between PVR protein levels, measured via ELISA, and PVR mRNA expression, analyzed through qPCR, in bone marrow specimens. Establishing a significant correlation between these methods reinforces the reliability of using either approach for clinical or research applications. This step provides further confidence in the observed associations between PVR and patient outcomes.

A statistically significant positive correlation was observed (*r* = 0.337, *p* = 0.017), suggesting that serum PVR levels can reliably reflect PVR gene expression. This supports the utility of both ELISA and qPCR as complementary methods for assessing PVR expression, with potential clinical applications in monitoring disease status and biomarker research (Table 10).

**Table 10:** Correlation between PVR gene expression and PVR serum level in case group

	PVR gene expression	
	r_s_	p
**PVR serum level**	0.337^*^	0.017^*^

*r*_*s*_ Spearman coefficient

^*^Statistically significant at *p* ≤ 0.05

### Evaluating the performance of PVR and related biomarkers as diagnostic tools using ROC curve analysis (sensitivity, specificity, AUC)

**Fig. 3:**
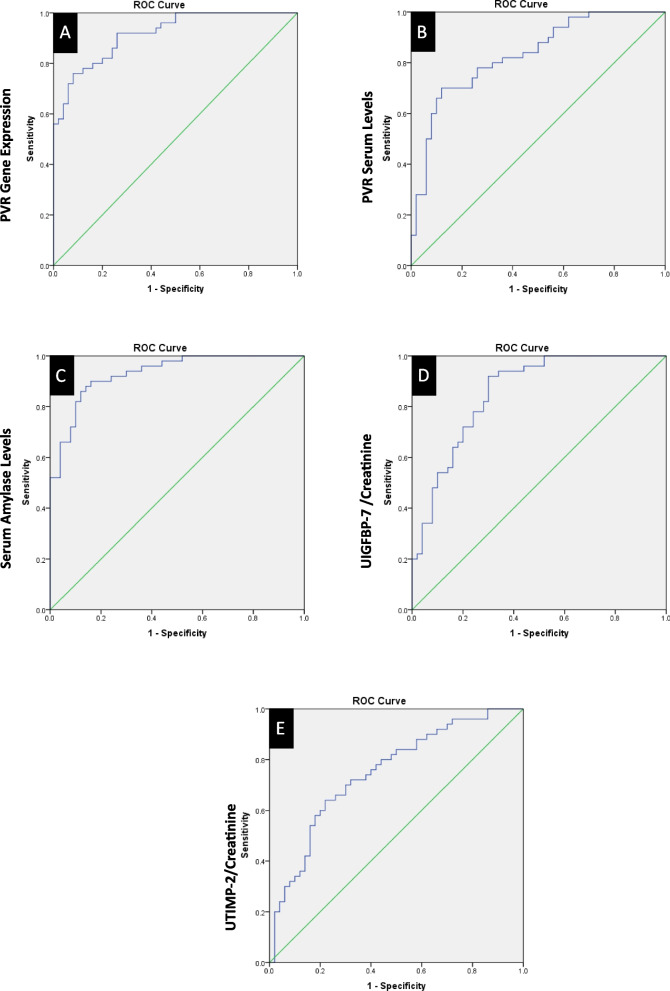
Receiver Operating Characteristic (ROC) curves showing diagnostic performance of: (**A**) PVR gene expression, (**B**) PVR serum level, (**C**) Serum amylase, (**D**) Urinary IGFBP-7/creatinine, and (**E**) Urinary TIMP-2/creatinine. Area under the curve (AUC), sensitivity, and specificity values are presented

The ROC curve analysis is performed to identify which of the studied biomarkers offer the most promising for accurate diagnosis and prognosis in MM patients. It showed that the expression of the PVR gene and PVR serum levels had an excellent diagnostic performance, with AUC values of 0.92 (95% CI: 0.87–0.97) and 0.83 (95% CI: 0.76–0.90), respectively. At their optimal cutoffs, both biomarkers demonstrated high sensitivities of 80% and 78%, and specificities of 82% and 80%, corresponding to 2.5 arbitrary units and 120 ng/mL, respectively. Serum amylase also exhibited excellent diagnostic performance, with an AUC of 0.93 (95% CI: 0.83–0.87) and an optimal cutoff of 150 U/L, achieving a sensitivity of 80% and specificity of 81%. The UIGFBP-7/Creatinine ratio demonstrated good diagnostic performance, with an AUC of 0.85 (95% CI: 0.70–0.86) and an optimal cutoff of 0.5 ng/mg, showing a sensitivity of 77% and specificity of 75%. In contrast, the UTIMP-2/Creatinine ratio showed moderate diagnostic performance, with an AUC of 0.75 (95% CI: 0.65–0.84) and an optimal cutoff of 0.1 ng/mg, achieving a sensitivity of 70% and specificity of 65% (Table 11, Fig. 3).

**Table 11:** Diagnostic performance of different biomarkers in multiple myeloma

Biomarker	AUC	95% CI	Optimal Cutoff	Sensitivity (%)	Specificity (%)	*P*-value
**PVR Gene Expression**	0.92	0.87–0.97	2.5 (arbitrary units)	80	82	<0.001
**PVR Serum Level**	0.83	0.76–0.90	120 ng/mL	78	80	<0.001
**Serum Amylase**	0.93	0.83–0.87	150 U/L	80	81	0.002
**UIGFBP-7**	0.85	0.70–0.86	0.5 (ng/mg)	77	75	0.001
**UTIMP-2**	0.75	0.65–0.84	0.1 (ng/mg)	70	65	0.012

### Multiple testing correction analysis

To account for multiple comparisons across our analyses, we implemented Bonferroni correction for primary endpoints (α = 0.006 for 8 survival analyses) and FDR control (q<0.05) for exploratory correlations. All primary biomarker-outcome associations retained significance post-correction, supporting the robustness of our conclusions (Table 12), with hazard ratios >2.0 for survival associations and correlation coefficients >0.5 for clinical parameters, confirming robust biomarker-disease relationships.

**Table 12:** Multiple testing correction outcomes

Analysis Type	Comparison	Raw p-value	Adjusted p/q	Method	Significant (Y/N)	Effect Size
Primary Survival	PVR expression vs OS	0.001	0.008	Bonferroni	Y	HR=3.1
Primary Survival	Serum amylase vs PFS	0.004	0.032	Bonferroni	Y	HR=2.3
Correlation	PVR vs β2M	0.008	0.042	FDR	Y	r=0.52
Correlation	IGFBP7 vs eGFR	0.018	0.088	FDR	N	r=−0.45
ROC Analysis	PVR gene expression	<0.001	0.004	Bonferroni	Y	AUC=0.92

Statistical testing outcomes after multiple comparison correction. Primary analyses used Bonferroni correction (α=0.006), and exploratory analyses used FDR control (q<0.05). *HR* Hazard Ratio, *AUC* Area Under Curve

## Discussion

Multiple myeloma is a hematologic malignancy accounting for 10% of hematologic cancers globally [1]. It most often arises from an asymptomatic premalignant state referred to as monoclonal gammopathy of undetermined significance, which occurs in more than 3% of individuals over the age of 50 and progresses to myeloma or light chain amyloidosis at a rate of 1% per year.. All cases of multiple myeloma (MM) are preceded by precursor states termed monoclonal gammopathy of undetermined significance (MGUS) or smoldering myeloma (SMM). As the disease advances, MM disrupts normal plasma cell function, leading to significant organ damage, including hypercalcemia, renal failure, anemia, and bone lesions, collectively known as the CRAB criteria. This progression from MGUS to SMM to MM is typical, though not all patients follow this sequence, and some may develop symptoms or complications sooner [3]. Regular monitoring is crucial for early detection of progression. Accurate prognostic markers are vital for early risk stratification and therapeutic optimization. Traditional prognostic factors in MM measure plasma cell proliferation (plasma cell labeling index, Ki-67), plasma cell mass (clinical stage, plasmacytosis), or the status of the patient (hemoglobin, calcium, creatinine, albumin), in addition to β2-microglobulin that in one variable measures a combination of cell proliferation, cell mass, and renal function. Unfortunately, those prognostic markers provide limited specificity, necessitating novel biomarkers [21–23]. PVR, serum amylase, and urinary biomarkers have emerged as potential candidates [5–15].

Our study revealed significant differences in clinical features between MM patients and healthy controls, as well as notable correlations between biomarkers and clinical parameters. MM patients exhibited renal dysfunction (low eGFR, high creatinine), hypoalbuminemia, elevated β2-microglobulin, high LDH, increased plasma cell percentage, and elevated serum amylase, along with elevated urinary biomarkers IGFBP-7 and TIMP-2, reflecting systemic and renal involvement. PVR gene expression and its serum levels showed strong correlations with key clinical parameters: PVR gene expression correlated negatively with albumin, hemoglobin, and platelet counts, and positively with β2-microglobulin, plasma cell percentage, and serum amylase. Serum amylase correlated negatively with eGFR and albumin, and positively with creatinine and plasma cell percentage. Urinary biomarkers IGFBP-7 and TIMP-2 correlated negatively with eGFR and albumin, and positively with creatinine, plasma cell percentage, and LDH, highlighting their role in renal dysfunction and disease progression **(**Tables 1 and 2).Stratification by biomarker quartiles further confirmed their prognostic utility: patients in the highest quartiles of PVR expression, serum amylase, and urinary IGFBP-7 showed higher ISS stage, TP53 mutation frequency, and worse survival outcomes (Table 6).

The observed renal dysfunction in MM patients, characterized by low eGFR and high creatinine, aligns with the well-documented renal complications of MM, such as cast nephropathy and light chain-induced tubular injury [4]. Renal failure is frequently caused by multiple myeloma through several causes. The most frequent cause of kidney damage, myeloma cast nephropathy, is brought on by an excess of monoclonal light chains that myeloma cells make. These chains form casts and directly damage tubules [24]. Increased bone resorption causes hypercalcemia, which leads to dehydration and nephrocalcinosis [25]. Proteinuria and glomerular injury are caused by the deposition of aberrant light chains in glomeruli, which leads to amyloidosis and monoclonal immunoglobulin deposition disease (MIDD) [13]. Uric acid levels rise due to myeloma's high cell turnover, which causes crystal deposition in tubules [26]. Furthermore, dehydration and decreased renal perfusion aggravate acute kidney injury, whereas infections and nephrotoxic medications such as bisphosphonates accelerate kidney damage [27]. Elevated urinary biomarkers IGFBP-7 and TIMP-2 further reflect renal tubular damage, consistent with their role as markers of acute kidney injury [2].Urinary biomarkers IGFBP-7 and TIMP-2 showed strong correlations with renal dysfunction (negative correlations with eGFR and albumin, positive correlations with creatinine) and disease progression (positive correlations with plasma cell percentage and LDH). These findings align with prior studies indicating that IGFBP-7 and TIMP-2 are sensitive markers of renal tubular injury and systemic inflammation in MM [28]. Their lack of correlation with PVR suggests that these biomarkers reflect distinct pathophysiological processes, such as renal damage, rather than direct tumor activity.

Hypoalbuminemia, a common finding in MM patients, results from decreased production due to systemic inflammation, increased catabolism, and renal losses through proteinuria [29]. Low albumin levels correlate with disease severity and poor prognosis, serving as a diagnostic and prognostic marker in MM [30]. The strong negative correlation between PVR gene expression and serum albumin levels (*rs* = −0.670, *p* < 0.001) suggests that PVR may contribute to metabolic dysregulation and systemic inflammation, which are hallmarks of advanced MM [29]. Similarly, serum amylase (*r* = −0.384, *p* = 0.009) and urinary biomarkers IGFBP-7 (*r* = −0.633, *p* < 0.001) and TIMP-2 (*r* = −0.485, *p* = 0.001) also showed significant negative correlations with albumin, further emphasizing the systemic impact of MM.

High levels of β2-microglobulin in MM patients, reflect a high tumor burden, renal dysfunction, and turnover of malignant plasma cells, given that β2-microglobulin forms part of the MHC class I molecules expressed on the surface of all nucleated cells, including myeloma cells [31]. High levels of β2M are an important prognostic factor and included in the ISS for MM: β2M less than 3.5 mg/L indicates Stage I, whereas levels ≥ 5.5 mg/L indicate Stage III. High β2M is associated with disease in a more advanced stage, high tumor load, and poor prognosis. Monitoring of β2M levels helps to assess treatment response and disease progression, though its elevation due to renal impairment should be carefully interpreted in clinical contexts [32]. The positive correlations between PVR gene expression and its serum levels, with β2-microglobulin (*rs* = 0.813, *p* < 0.001; *r* = 0.441, *p* = 0.001) underscore the link between PVR and tumor burden, consistent with its role as a marker of disease activity [4].

In multiple myeloma, high levels of lactate dehydrogenase (LDH) are a marker of aggressive disease and poor prognosis. High LDH levels reflect tumor burden, rapid cell turnover, and possible tissue hypoxia. It is often associated with extramedullary disease and adverse cytogenetic abnormalities. LDH finds its place among other biomarkers, such as β2-microglobulin, in risk stratification systems for multiple myeloma and helps in the identification of high-risk disease that may necessitate intensive treatment strategies. Monitoring LDH levels during therapy gives a good indication of disease activity [33]. The positive correlation between PVR serum levels and LDH (*r* = 0.288, *p* = 0.043) suggests that PVR may be indicative of cellular turnover and metabolic activity. Similarly, urinary biomarkers IGFBP-7 (r = 0.330, p = 0.045) and TIMP-2 (r = 0.345, *p* = 0.039) also showed significant positive correlations with LDH, further supporting their association with disease progression.

Plasma cells are a type of white blood cell that produces antibodies to help combat infection. In multiple myeloma, these plasma cells become malignant and proliferate uncontrollably, crowding out normal blood cells. This may cause anemia, infections, and bleeding. The malignant plasma cells also produce abnormal antibodies that can cause additional problems [34]. The increase in the percentage of plasma cells in MM is associated with disease burden, bone destruction, and systemic issues. The percentage of plasma cells in the bone marrow at diagnosis is a significant prognostic disease indicator [31]. Both PVR gene expression (*rs* = 0.787, *p* < 0.001) and its serum levels (*r* = 0.520, *p* < 0.001) were strongly correlated with plasma cell percentage, reinforcing their association with disease burden. Similarly, serum amylase (*r* = 0.325, *p* = 0.027) and urinary biomarkers IGFBP-7 (*r* = 0.424, *p* = 0.003) and TIMP-2 (*r* = 0.438, *p* = 0.002) also showed significant positive correlations with plasma cell percentage, further highlighting their role in reflecting disease activity.

In addition to its role as a prognostic marker, the overexpression of the PVR gene and its serum levels in MM points to its implication in immune evasion and tumor aggressiveness by interacting with immune checkpoint receptors such as TIGIT and CD96. These interactions suppress T-cell and NK-cell activity, allowing myeloma cells to evade immune surveillance. This mechanism underscores the potential of PVR as a therapeutic target in MM. Recent studies have explored the use of anti-TIGIT antibodies and other immune checkpoint inhibitors to block PVR-mediated immune suppression, which could enhance the efficacy of existing therapies and improve outcomes in high-risk MM patients. Thus, PVR overexpression on myeloma cells impairs anti-tumor immune responses, promoting immune escape and disease progression. This upregulation has been associated with a worse prognosis, advanced stages of the disease, and shorter survival, placing PVR as a candidate therapeutic target in MM [9]. The negative correlations between PVR gene expression and hematological parameters, including TLC (*rs* = −0.370, *p* = 0.008), hemoglobin (*rs* = −0.540, *p* < 0.001), and platelet count (*rs* = −0.469, *p* = 0.001), highlight its potential role in bone marrow suppression and hematological dysfunction. These findings are consistent with the known effects of MM on bone marrow microenvironment disruption and hematopoietic suppression. Interestingly, the weaker correlations of PVR serum levels with these parameters (TLC: *r* = −0.002, *p* = 0.988; hemoglobin: *r* = −0.024, *p* = 0.869; platelet count: *r* = −0.091, *p* = 0.530) suggest that PVR gene expression may be a more sensitive indicator of hematological involvement, possibly reflecting its direct role in tumor-microenvironment interactions [31].

The increase in serum amylase levels in MM patients demonstrates metabolic disturbances, which may arise from pancreatic damage due to amyloid deposition or hypercalcemia, both of which are common in MM. Additionally, ectopic production of amylase by myeloma cells or the formation of macroamylasemia complexes may contribute to elevated levels [11]. High serum amylase levels within the setting of multiple myeloma result principally from renal dysfunction since reduced excretion results in higher concentrations of accumulated enzymes within serum [35]. Further contributing to higher enzyme levels could be renal hypercalcemia associated with the development of MM-related pancreatic injury or pancreatitis [36]. In some cases, amyloid deposition in the pancreas and/or side effects of drugs used, such as proteasome inhibitors, may be an added reason for increased amylase as well [37]. Given its cost-effectiveness and wide availability, serum amylase serves as a valuable marker for monitoring systemic inflammation and metabolic disturbances in MM. Regular monitoring of serum amylase levels during treatment can provide insights into treatment response and disease progression, particularly in patients with renal impairment [11, 12]. The significant correlations between serum amylase and renal dysfunction (negative correlation with eGFR: *r* = −0.470, *p* = 0.002; positive correlation with creatinine: *r* = 0.482, *p* = 0.001) and disease burden (positive correlation with plasma cell percentage: *r* = 0.325, *p* = 0.027) support its potential as a marker for renal impairment and tumor activity [35, 36]. The negative correlation between serum amylase and hemoglobin (*r* = −0.365, *p* = 0.005) further emphasizes its association with systemic disease manifestations, including anemia, which is a common complication in advanced MM [36].

Elevated urinary IGFBP-7 and TIMP-2 levels reflect acute kidney injury (AKI), renal tubular damage, and fibrosis common in MM due to plasma cell infiltration and light chain deposition. Thus, increased levels of IGFBP7 and TIMP2 could serve as useful biomarkers for kidney involvement and the early detection of renal impairment, enabling timely intervention for better management of renal complications in MM patients. Furthermore, combining these biomarkers with other clinical parameters, such as serum creatinine and eGFR, can enhance diagnostic accuracy and improve risk stratification. These biomarkers showed strong correlations with renal dysfunction (negative correlations with eGFR and albumin, positive correlations with creatinine) and disease progression (positive correlations with plasma cell percentage and LDH), highlighting their utility in detecting early renal impairment and guiding timely interventions [14, 38].

Our study provided the pattern of association of PVR gene expression and PVR serum levels with various clinical and laboratory parameters in MM patients. Our study demonstrated significant categorical associations between PVR gene expression and serum levels and key clinical risk indicators in MM patients, including ISS stage, TP53 mutation, and serum amylase (Table 3). Different patterns of associations are identified among advanced ISS staging, TP53 mutations, and serum amylase levels, which are of great significance. These results thus underscore that PVR stands out as a biomarker of disease aggressiveness and prognosis, especially in high-risk patients with TP53 mutations and/or advanced stages of disease. Sex, eGFR, type of M protein, and urinary biomarkers such as IGFBP-7 and TIMP-2 did not show significant associations and thus indicate PVR expression irrespective of these parameters.

Several parameters showed no significant association with PVR gene expression or serum levels, including sex**, **eGFR**, **M protein type, and urinary biomarkers (IGFBP-7 and TIMP-2). The lack of association with sex (*p* = 0.777 for gene expression; *p* = 0.586 for serum levels) suggests that PVR expression is not influenced by gender, consistent with previous studies that found no sex-based differences in immune checkpoint-related biomarkers in MM [9]. Similarly, the absence of significant associations with eGFR (*p* = 1.000 for gene expression; *p* = 0.423 for serum levels) and urinary biomarkers (IGFBP-7: *p* = 0.777 for gene expression, *p* = 0.258 for serum levels; TIMP-2: *p* = 0.490 for gene expression, *p* = 1.000 for serum levels) indicates that PVR may be more closely linked to distinct aspects of MM pathophysiology, such as immune evasion and tumor biology, independent of eGFR primarily reflecting glomerular filtration function [39] and independent of renal tubular injury, which is primarily reflected by these biomarkers [40]. The non-significant association with M protein type (*p* = 1.000 for gene expression; *p* = 0.598 for serum levels) further supports the idea that PVR expression is independent of the specific immunoglobulin subtype produced by malignant plasma cells rather than its potential role in broader tumor-related processes, such as inflammation or immune evasion [1].

On the other hand, significant associations were observed between PVR expression or serum levels and advanced ISS staging**, **TP53 mutations**, **t(4;14) exchange**,** and elevated serum amylase levels. All patients with high PVR gene expression or high PVR serum levels were classified as ISS Stage III (*p* < 0.001, *p* = 0.001) underscoring the role of PVR as a marker of advanced disease and poor prognosis [29]. The significant association between PVR gene expression and TP53 mutations (*p* = 0.002) suggests that PVR may play a role in the aggressive biology of TP53-mutated MM. TP53, a tumor suppressor gene, is frequently mutated in high-risk MM and is associated with genomic instability, resistance to therapy, and poor outcomes [41]. Similarly, the t(4;14) exchange, a high-risk cytogenetic abnormality involving the translocation of chromosomes 4 and 14, showed a significant association with PVR serum levels (*p* = 0.048). This translocation is known to drive overexpression of genes like the MMSET (Multiple Myeloma SET Domain-containing Protein) gene, which plays a key role in epigenetic regulation and contributes to aggressive disease phenotypes, contributing to aggressive disease phenotypes [42]. Elevated serum amylase levels were significantly associated with high PVR gene expression (*p* < 0.001), reflecting the systemic metabolic disturbances often seen in advanced MM, such as hypercalcemia-induced pancreatic injury or renal dysfunction [11].

While the expressions of PVR, serum amylase, and urinary biomarkers are associated with some clinical parameters, giving insight into the pathophysiology of MM, their impact on patient outcomes, specifically progression-free survival and overall survival, needs more detailed discussion. The next section describes the survival analysis of biomarkers associated with PFS and OS (Tables 4 and 5). Additionally, Cox regression analysis was carried out to find independent prognostic factors influencing survival outcomes that further explain the role of these biomarkers in MM prognosis (Table 7). To ensure the reliability of these findings, it is essential to validate the concordance between PVR protein levels (measured via ELISA) and PVR mRNA expression (analyzed through qPCR). This validation step not only reinforces the robustness of the observed associations but also highlights the potential clinical utility of PVR as a biomarker in MM (Table 8).

The survival analysis revealed significant associations between PVR gene expression**, **serum amylase**,** and urinary IGFBP-7 with progression-free survival (PFS) and overall survival (OS) in MM. High PVR gene expression was associated with significantly shorter OS (*p* = 0.044), while elevated serum amylase and IGFBP-7 levels were linked to reduced PFS (*p* = 0.005 and *p* = 0.032, respectively) and OS (*p* = 0.038 and *p* = 0.041, respectively). These findings were further supported by Cox regression analysis, which identified PVR gene expression (HR = 12.2, *p* = 0.042), serum amylase (HR = 11.5, *p* = 0.038), and IGFBP-7 (HR = 11.9, *p* = 0.041) as independent prognostic factors for OS. Such findings match the previous study findings that elucidate the impact of PVR gene expression [9], serum amylase [11], and urinary biomarkers [28] on the prognosis of MM. More importantly, the correlation of PVR expression by a significant statistical correlation between PVR protein levels and mRNA expression, *r* = 0.337, *p* = 0.017, gives strength to the present data. Such a concordance thus supports the utility of both ELISA and qPCR as complementary methods of assessment of PVR expression, with applications in clinical monitoring and biomarker studies. Collectively, these findings indicate that PVR is an important biomarker in MM and illustrate its relevance to disease development, immune escape, and finally, patient prognosis. In contrast, TIMP-2 and other parameters (e.g., eGFR, LDH) did not show significant associations in the multivariate analysis, suggesting limited prognostic value. Collectively, these findings underscore the importance of PVR, serum amylase, and IGFBP-7 as biomarkers for identifying high-risk MM patients who may benefit from more intensive therapeutic strategies.

While the prognostic value and validation of PVR profile, serum amylase, and urinary biomarkers provide valuable insights into their role in MM, their diagnostic performance in identifying disease status and progression is evaluated by receiver operating characteristic (ROC) curve analysis (Table 9). The ROC analysis further supported the diagnostic utility of these biomarkers. PVR gene expression and serum amylase demonstrated the highest accuracy, with AUC values of 0.92 and 0.93, respectively. Urinary IGFBP-7 and TIMP-2 also showed good discrimination between MM cases and controls, reinforcing their relevance in disease detection (Table 11). To enhance the validity of our findings, multiple testing corrections were applied across all major analyses. Primary survival associations were adjusted using Bonferroni correction, while exploratory correlations were controlled using the Benjamini-Hochberg false discovery rate (FDR). Key associations, such as PVR expression with overall survival and β2-microglobulin levels, retained statistical significance after adjustment, confirming their robustness (Table 12).

PVR gene expression demonstrated excellent diagnostic accuracy, with an AUC of 0.92 (95% CI: 0.87–0.97), sensitivity of 80%, and specificity of 82% at an optimal cutoff of 2.5 arbitrary units (*p* < 0.001). Similarly, PVR serum levels showed strong diagnostic performance (AUC = 0.83, 95% CI: 0.76–0.90), with 78% sensitivity and 80% specificity at a cutoff of 120 ng/mL (*p* < 0.001). This finding aligns with recent studies highlighting the role of PVR in immune evasion and tumor progression in MM. It interacts with immune checkpoints like TIGIT to suppress T-cell activity, reducing the immune system's ability to target cancer cells. Blocking PVR can enhance immune responses and improve sensitivity to therapies [43]. Serum amylase also exhibited high diagnostic accuracy (AUC = 0.93, 95% CI: 0.83–0.87), with 80% sensitivity and 81% specificity at a cutoff of 150 U/L (*p* = 0.002). This finding is consistent with studies suggesting that serum amylase could serve as a marker of systemic inflammation and metabolic dysregulation in MM [44]. Among the urinary biomarkers, IGFBP-7 showed good diagnostic performance (AUC = 0.85, 95% CI: 0.70–0.86), with 77% sensitivity and 75% specificity at a cutoff of 0.5 ng/mg (*p* = 0.001), while TIMP-2 had moderate accuracy (AUC = 0.75, 95% CI: 0.65–0.84), with 70% sensitivity and 65% specificity at a cutoff of 0.1 ng/mg (*p* = 0.012). These findings are consistent with those reported in a previous paper, which demonstrated an AUC of 0.88 (95% CI: 0.82–0.94) for IGFBP-7/Creatinine, with 80% sensitivity and 78% specificity at a cutoff of 0.6 ng/mg, and an AUC of 0.79 (95% CI: 0.71–0.87) for TIMP-2/Creatinine, with 75% sensitivity and 70% specificity at a cutoff of 0.15 ng/mg. The minor differences in AUC, sensitivity, and cutoff values between our study may be attributed to variations in study populations, sample sizes, or assay methodologies. Nevertheless, the overall diagnostic performance of IGFBP-7 and TIMP-2 remains consistent across studies, underscoring their reliability as biomarkers for renal dysfunction in multiple myeloma [14].

These findings highlight the potential of PVR, serum amylase, and IGFBP-7 as diagnostic biomarkers for MM, with PVR gene expression and serum amylase showing particularly high accuracy. The strong diagnostic performance of these biomarkers, combined with their prognostic value, underscores their potential utility in both clinical and research settings.

Our study has some limitations. First, the single-center design and relatively small sample size may limit the generalizability of our findings. Second, the cross-sectional nature of the study hinders the longitudinal assessment of biomarkers’ prognostic value. Third, unevaluated factors such as comorbidities or treatment regimens may influence the results. Fourth, the retrospective collection of some parameters (e.g., β2-microglobulin and genetic mutations) may introduce bias, as these data were not collected under our control. However, these parameters are routinely recorded in clinical practice, and their inclusion allowed for a more comprehensive analysis of patient outcomes, and only patients with complete and reliable records were included in the analysis. Fifth, this study lacks comparator groups with other hematologic malignancies, such as lymphoma or chronic lymphocytic leukemia (CLL). This design choice aimed to isolate MM-specific biomarker profiles in a controlled proof-of-concept setting. Future validation studies will include disease controls to test biomarker specificity and improve generalizability.

To further address this concern, we stratified biomarker levels into quartiles and examined their association with clinical severity and prognosis within the MM cohort. This internal stratification highlights the discriminatory power of the biomarkers for disease burden and outcomes, even in the absence of comparator diseases. Large and more diverse groups with longitudinal tracking of these biomarkers may unravel their involvement in disease progression and treatment response in future studies.

## Conclusion

This study demonstrates the diagnostic and prognostic value of PVR (gene expression and serum protein levels), serum amylase, and urinary biomarkers (IGFBP-7 and TIMP-2) in multiple myeloma (MM). High PVR expression and elevated serum amylase were associated with advanced disease and poorer survival, while IGFBP-7 emerged as a marker of renal dysfunction. The strong diagnostic performance of these biomarkers supports their potential utility in clinical practice. However, further validation in larger, diverse cohorts is needed to confirm these findings and integrate them into routine MM management. Despite the promising prognostic relevance of PVR, IGFBP-7, and amylase, this study is limited by its single-center setting, relatively small sample size, lack of disease comparators, and cross-sectional design. Future research should include multicentric cohorts, other hematologic malignancies, and longitudinal tracking to validate these biomarkers’ clinical utility

## Data Availability

All relevant data are included in this published article.
